# The Experimental TASK-1 Potassium Channel Inhibitor A293 Can Be Employed for Rhythm Control of Persistent Atrial Fibrillation in a Translational Large Animal Model

**DOI:** 10.3389/fphys.2020.629421

**Published:** 2021-01-21

**Authors:** Felix Wiedmann, Christoph Beyersdorf, Xiao-Bo Zhou, Manuel Kraft, Kathrin I. Foerster, Ibrahim El-Battrawy, Siegfried Lang, Martin Borggrefe, Walter E. Haefeli, Norbert Frey, Constanze Schmidt

**Affiliations:** ^1^Department of Cardiology, Heidelberg University, Heidelberg, Germany; ^2^DZHK (German Center for Cardiovascular Research), Partner Site Heidelberg/Mannheim, Heidelberg University, Heidelberg, Germany; ^3^HCR, Heidelberg Center for Heart Rhythm Disorders, Heidelberg University, Heidelberg, Germany; ^4^First Department of Medicine, University Medical Center, Mannheim University, Mannheim, Germany; ^5^Department of Clinical Pharmacology and Pharmacoepidemiology, Heidelberg University, Heidelberg, Germany

**Keywords:** A293, antiarrhythmic pharmacotherapy, atrial fibrillation, cardioversion, KCNK3, TASK-1

## Abstract

**Background:**

Upregulation of the two-pore-domain potassium channel TASK-1 (hK_2__*P*_3.1) was recently described in patients suffering from atrial fibrillation (AF) and resulted in shortening of the atrial action potential. In the human heart, TASK-1 channels facilitate repolarization and are specifically expressed in the atria. In the present study, we tested the antiarrhythmic effects of the experimental ion channel inhibitor A293 that is highly affine for TASK-1 in a porcine large animal model of persistent AF.

**Methods:**

Persistent AF was induced in German landrace pigs by right atrial burst stimulation via implanted pacemakers using a biofeedback algorithm over 14 days. Electrophysiological and echocardiographic investigations were performed before and after the pharmacological treatment period. A293 was intravenously administered once per day. After a treatment period of 14 days, atrial cardiomyocytes were isolated for patch clamp measurements of currents and atrial action potentials. Hemodynamic consequences of TASK-1 inhibition were measured upon acute A293 treatment.

**Results:**

In animals with persistent AF, the A293 treatment significantly reduced the AF burden (6.5% vs. 95%; *P* < 0.001). Intracardiac electrophysiological investigations showed that the atrial effective refractory period was prolonged in A293 treated study animals, whereas, the QRS width, QT interval, and ventricular effective refractory periods remained unchanged. A293 treatment reduced the upregulation of the TASK-1 current as well as the shortening of the action potential duration caused by AF. No central nervous side effects were observed. A mild but significant increase in pulmonary artery pressure was observed upon acute TASK-1 inhibition.

**Conclusion:**

Pharmacological inhibition of atrial TASK-1 currents exerts *in vivo* antiarrhythmic effects that can be employed for rhythm control in a porcine model of persistent AF. Care has to be taken as TASK-1 inhibition may increase pulmonary artery pressure levels.

## Introduction

Atrial fibrillation (AF) represents a major clinical and socio-economic burden (Hindricks et al., 2020). With about 2–4 percent of the western population suffering from AF, it is by far the most common sustained arrhythmia (Hindricks et al., 2020). Occurrence of AF is strongly correlated with age. Therefore, it can be expected that demographic changes will result in an increased incidence and prevalence of AF. Current therapeutic strategies consist of pharmacological, interventional, and surgical approaches and often show suboptimal effectiveness (January et al., 2019). Novel mechanism-based therapeutic approaches might improve the effectiveness and safety of pharmacologic AF treatment (Peyronnet and Ravens, 2019). In this context, it will be important to find an atrial-selective therapeutic target for AF therapy.

The two-pore-domain potassium (K_2P–_) channels represent the most recently characterized group of potassium channels (Goldstein et al., 2001). Their 15 members are abundantly expressed throughout the human body and contribute to various physiological processes, including the regulation of cardiac rhythm, blood pressure, neuroprotection and apoptosis (Patel and Honore, 2001; Bayliss and Barrett, 2008; Lloyd et al., 2011; Schmidt et al., 2013, 2015, 2017; Wiedmann et al., 2020b).

TASK-1 (TWIK [tandem of P domains in a weak inward rectifying K^+^ channel]-related acid sensitive K^+^ channel; K_2__*P*_3.1) is a member of the K_2__*P*_-family that shows atrial-specific expression in the human heart (Limberg et al., 2011; Rinné et al., 2015; Schmidt et al., 2015, 2017). It was recently shown that TASK-1 is upregulated in cardiomyocytes from patients suffering to chronic AF (cAF) (Schmidt et al., 2015, 2017). This results in a shortening of the action potential duration (APD), which represents a fundamental pathophysiological mechanism underlying AF (Nattel et al., 2008; Schotten et al., 2011). In human atrial cardiomyocytes isolated from AF patients, specific blockade of TASK-1 currents prolonged APD to physiological values (Schmidt et al., 2015; Schmidt et al., 2017). TASK-1 inhibition therefore represents a promising novel strategy for mechanism-based AF-therapy.

This preclinical study was designed to investigate whether specific pharmacological TASK-1 inhibition can be used for long-term rhythm control of persistent AF in a translationally relevant porcine AF model. The high-affinity TASK-1 inhibitor A293 was evaluated as an experimental anti-arrhythmic treatment. This aromatic carbonamide represents an experimental compound that is active in the nanomolar to micromolar range (Putzke et al., 2007b; Kiper et al., 2015; Schmidt et al., 2015; Wiedmann et al., 2019, 2020a). As TASK-1 channels are expressed in pulmonary artery (PA) smooth muscle cells where they might contribute to regulation of the vascular tone (Olschewski et al., 2006), hemodynamic effects of TASK-1 inhibition were studied upon acute TASK-1 channel inhibition.

## Materials and Methods

### Ethics Statement

All animal experiments have been carried out in accordance with the Guide for the Care and Use of Laboratory Animals as adopted and promulgated by the United States National Institutes of Health (NIH publication No. 86-23, revised 1985), with the EU Directive 2010/63/EU, and with the German Law on the Protection of Animals. Approval for experiments involving pigs was granted by the local Animal Welfare Committee (Regierungspräsidium Karlsruhe, Germany, reference numbers G-296/14 and G-217/18).

### Experimental Design

**FIGURE 1:**
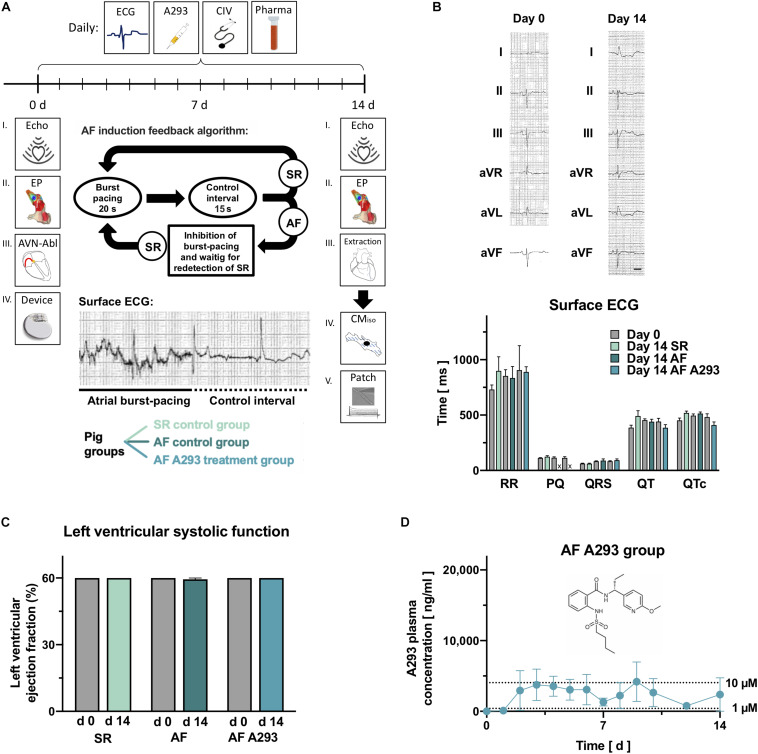
Experimental protocol, surface electrocardiograms and echocardiography parameters. **(A)** Subsequent to an electrophysiological (EP) investigation and pacemaker (device) implantation, *n* = 15 pigs were randomized to atrial fibrillation (AF) induction or a sinus rhythm (SR) control group (*n* = 5). Pigs randomized to the AF group received AV-node ablations to prevent development of tachycardia-induced heart failure. The AF group was divided into subgroups receiving i.v. sham (NaCl 0.9%; *n* = 5) or A293 treatment (1 mg/kg bodyweight per day; *n* = 5). During the 14 days treatment period, pigs received daily sham or A293 treatments, and electrocardiogram (ECG) recordings and clinical investigations (CIV) were performed, and blood samples were taken. At the end of the observation period, electrophysiological investigations were repeated and atrial cardiomyocytes were subjected to patch clamp measurements. The protocol of burst-pacing, applied by the implanted pacemakers is visualized as insert. **(B)** Representative surface ECG measurements, recorded in a SR control pig at days 0 and 14, are shown in the upper part. No significant differences were observed between the 3 groups when comparing RR and PQ intervals, QRS durations, QT and QTc intervals at day 0 and day 14 with Wilcoxon matched pairs single rank tests. No PQ intervals could be measured in animals which underwent AV node ablation. **(C)** Values of left ventricular ejection fraction, quantified with echocardiography at day 0 and day 14 did not change significantly (means of *n* = 4–5 animals; error bars, SEM; scale bar, 200 ms). **(D)** Plasma levels of A293 (trough levels) during the 14 days period, measured by mass spectrometry. The chemical structure of A293 is provided as insert. Please note that blood clots in central vein catheters caused a relevant loss of follow up at some time points (means of *n* = 1–5 animals; error bars, SEM).

Fifteen German landrace pigs of both sexes (30–40 kg bodyweight) were randomized to a total of three different groups (Figure 1A). Two reference groups with SR and AF (14 days SR, *n* = 5; 14 days AF, *n* = 5), which received sham treatment (NaCl 0.9%), and a treatment group with AF (14 days AF A293, *n* = 5), which received daily treatment with the high affinity TASK-1 inhibitor A293 to substantiate the hypothesis that anti-TASK-1 treatment would provide rhythm control in AF.

On day 0 of the 14 days experimental follow-up 12-lead ECG recording, transthoracic echocardiographic investigation of left ventricular function, electrophysiological (EP) study, dual chamber pacemaker implantation, and central venous catheter implantation was performed. An atrioventricular (AV) node ablation was performed in animals randomized to AF groups to prevent tachycardiomyopathy as a consequence of AF induction (Schmidt et al., 2019) and ventricular backup pacing was provided via the implanted pacemakers.

In addition to clinical examination and 4-lead surface ECG recording, A293 was applied intravenously (i.v.) daily during the follow-up period. On day 14 prior to final surgery 12-lead ECG recording, echocardiography, and EP study was repeated before euthanization and organ explantation. All surgical procedures and catheter interventions were performed in aseptic technique and a single dose of cefuroxime (750 mg, i.v.; Ratiopharm GmbH, Ulm, Germany) was given prophylactically pre-surgery.

No exclusion criteria were defined and all eligible animals were included in the study. During the follow-up period, pigs were fed restrictively twice a day with complete feeding stuffs (SAF 130M, ZG Raiffeisen, Karlsruhe, Germany) with water being provided *ad libitum*. A light-dark cycle of 12/12 h, a room temperature of 20°C ± 2°C and a maximum housing density according to directive 2010/63/EU in specific pathogen-free conditions was adhered to at all times. Environmental enrichment was provided with biting woods, chains, and feeding balls.

To further determine acute effects of TASK-1 inhibition on hemodynamic parameters 4 pigs of both sexes (30–40 kg bodyweight) were treated as described below.

### Anesthesia

Upon sedation with midazolam (1 mg/kg, intramuscular [i.m.]; Hameln Pharma Plus GmbH, Berlin, Germany), azaperon (5 mg/kg, i.m.; Elanco, Bad Homburg, Germany), and ketamine (10 mg/kg, i.m.; Zoetis Deutschland GmbH, Berling, Germany), a peripheral venous catheter was established and an initial bolus of propofol (1.5 mg/kg, i.v.; Fresenius Kabi, Bad Homburg Germany) and buprenorphine (0.02 mg/kg, i.v.; Bayer Vital GmbH Tiergesundheit, Leverkusen, Germany) was administered. Subsequently pigs were intubated and mechanically ventilated (Dräger Primus system; Dräger, Lübeck, Germany). Anesthesia was perpetuated with propofol (4–8 mg/kg/h, i.v.) and isoflurane (0.5–2 vol.%; Baxter Deutschland GmbH, Heidelberg, Germany). Of note, no isoflurane was administered before completion of EP measurements to avoid interference of volatile anesthetics with cardiac K_2__*P*_-channel function (Putzke et al., 2007a).

### Echocardiography

Pigs were echocardiographically characterized after sedation on day 0 and day 14 prior to surgery, respectively (Philips Healthcare Sonos 5500, Hamburg, Germany). AF was electrically converted to sinus rhythm (SR) before echocardiography examinations. End-diastolic and end-systolic volumes and segmental left ventricular wall motion was visually quantified from the apical 4-chamber and 2-chamber view to determine left ventricular ejection fraction.

### Electrophysiological Study

Intracardiac EP studies were performed after intubation on day 0 and day 14. If pigs remained in persistent AF after deactivation of burst stimulation, electric cardioversion was performed in deep anesthesia and EP studies were postponed for at least 30 min. After cannulation of the right jugular vein, two quadripolar catheters (Abbott Laboratories, Chicago, IL, United States) were placed in the high right atrium and the right ventricular apex under fluoroscopic guidance. A UHS 20 stimulus generator (Biotronic, Berlin, Germany) delivered intracardiac stimuli and the EP Lab duo system (Bard Electrophysiology Division, Lowell, MA, United States) was used for recording, analyzing, and storing of electrocardiograms (ECGs). Surface ECGs were recorded as 12-lead ECGs using conventional adhesive electrodes (3M red dot, 3M, Maplewood, MN, United States) in Einthoven/Goldberger/Wilson configuration. QT intervals were corrected using the Bazett’s formula (Bazett, 1920). Upon placement of the catheters, pacing thresholds were determined (0.5–2 V at 2.9 ms pulse width). Effective refractory periods (ERPs) were measured at twice the diastolic pacing threshold using a conditioning train of 9 basic stimuli (S1; 500, 400, or 300 ms as indicated) followed by an extra stimulus (S2) starting 50 ms after the expected ERP. Coupling intervals of extra stimuli were decreased in 5 ms decrements until refractoriness of the S2 stimulus was achieved. The shortest coupling interval eliciting a propagated atrial response was taken as the ERP. To measure sinus node recovery times (SNRTs), atrial simulation was applied at basic cycle lengths (BCLs) of 500–300 ms for 30 s and pre automatic pauses from the last stimulus to the first intrinsic sinus beat were measured. Corrected SNRTs (cSNRTs) were calculated by subtracting the intrinsic cycle length from the respective SNRT. Sinuatrial conduction time (SACT) was measured by applying the Narula et al. (1978) as well as the Strauss et al. (1973) methods.

### AV Node Ablation

Prior to pacemaker implantation and AF induction all animals randomized to the AF groups were subjected to AV node ablation to prevent development of tachycardia-induced heart failure, a common limitation of porcine AF models. AV node ablations were accomplished via radio frequency (RF) by a trained interventional electrophysiologist. Following venipuncture, a non-cooled 7F 4 mm tip ablation catheter (Cerablate, Osypka, Rheinfelden, Germany) connected to an Osypka HAT-300 RF generator was navigated to the AV node area under fluoroscopic and electrocardiographic guidance (see Supplementary Figures 1A,B). A permanent third grade AV block was usually achieved within 90 s of temperature controlled RF application (temperature limit 70°C) as shown in Supplementary Figures 1B,C. Autopsy during the final operation 14 days after AV node ablation showed circumscribed scars in the area of the compact AV node without relevant collateral damage (Supplementary Figure 1D).

### Porcine Atrial Fibrillation Model

Induction of AF in domestic pigs was carried out by episodes of rapid atrial burst pacing (40 Hz), delivered via an implanted cardiac pacemaker (St. Jude Medical, St. Paul, MN, United States). The pacemakers were programmed to a continuous loop of atrial burst stimulation (40 Hz) for 20 s followed by a 15 s interval without atrial burst stimulation to assess the atrial rhythm. Upon detection of continuing AF episodes, burst stimulation was paused. Whenever the pigs returned to SR for >15 s, the episodes of burst pacing were resumed as describes above.

During the 14 days follow-up period, animals were clinically examined and 4-lead surface ECGs (60 s of recording per ECG) were recorded once per day. Burst pacing was continued during the ECG measurements, and animals were awake and alert at a constant level. For quantification of the pig’s individual AF burden, the rhythm during the 15 s off-pacing intervals was analyzed in each daily 60 s ECG recording. Atrial rhythm was independently assessed by two cardiologists blinded to the study group. The AF burden was calculated according to the equation: [number of daily surface ECGs with AF]/[cumulative number of surface ECG recordings].

### Drug Administration

In the pharmacological treatment group, a central venous catheter was implanted on day 0 for daily drug administration. After preparation and cannulation of the left jugular vein the catheter’s outer end was subcutaneously tunneled to the neck of the pig to avoid manipulation and to lower the risk of infections. The aromatic carbonamide high-affinity TASK-1 inhibitor A293, 2-(butylsulfonylamino)-N-[(1R)-1-(6-methoxy-3-3pyridyl)propyl]-benzamide, was applied daily in a dose of 1 mg/kg bodyweight as short infusion (100 ml 0.9% NaCl) over 5 min. A293 was synthesized by ChiroBlock (ChiroBlock, Wolfen, Germany) with a purity of 98% and dissolved in dimethyl sulfoxide (DMSO) to a concentration of 100 mM. Stock solutions were stored at −20°C. Before drug application, plasma samples were drawn for assessment of trough levels of A293.

### Hemodynamic Measurements

Four pigs of both sexes (30 to 40 kg bodyweight) were anesthetized, intubated and mechanically ventilated as described above. Femoral artery and jugular veins were cannulated for invasive measurement of blood pressure levels (Dräger infinity system) and a Swan-Ganz catheter (Biosensors International, Morges, Switzerland) was employed for measurement of the PA pressure. Under continuous intravenous propofol infusion A293 1 mg/kg bodyweight was administered as short infusion (100 ml 0.9% NaCl) over 5 min and hemodynamic parameters were compared to values obtain under control conditions.

### Tissue Handling and Porcine Atrial Cardiomyocyte Isolation

Porcine hearts were explanted at the end of final surgery after euthanization by i.v. injection of potassium chloride in deep anesthesia. Tissue samples of the anterior right atrium were immediately transferred into preoxygenated Ca^2+^-free Tyrode’s solution [100 mM NaCl, 10 mM KCl, 1.2 mM KH_2_PO_4_, 5 mM MgSO_4_, 50 mM taurine, 5 mM 3-(N-morpholino) propanesulfonic acid (MOPS) and 20 mM glucose, pH 7.0 with NaOH] supplemented with 2,3-butanedione monoxime (BDM, 30 mM; Sigma-Aldrich, St. Louis, MO, United States). Isolation of single atrial cardiomyocytes was performed as previously described (Schmidt et al., 2015). Atrial tissue samples were rinsed three times for 3 min in Ca^2+^-free Tyrode’s solution. The solutions were preoxygenated with 100% O_2_ at 37°C. Subsequently aliquots were digested with collagenase type I (288 U/ml; Worthington) and protease type XXIV (5 mg/ml; Sigma Aldrich) for 15 min before Ca^2+^ concentration was increased to 0.2 mM and samples were stirred for another 35 min in protease-free solution. Finally, single rod-shaped cardiomyocytes could be obtained and resuspended in storage medium [20 mM KCl, 10 mM KH_2_PO_4_, 10 mM glucose, 70 mM K glutamate, 10 mM β-hydroxybutyrate, 10 mM taurine, 10 mM ethylene glycol tetraacetic acid (EGTA), and 1% albumin] prior to electrophysiological characterization in patch clamp experiments.

### Patch Clamp Electrophysiology

Patch clamp pipettes were pulled from borosilicate glass (1B120F-4; World Precision Instruments, Berlin, Germany) and filled with patch clamp internal solution [60 mM KCl, 65 mM K glutamate, 3 mM K_2_ATP, 0.2 mM Na_2_GTP, 2 mM MgCl_2_ 5 mM EGTA, and 5 mM 4-(2-hydroxyethyl)piperazine-1-ethanesulfonic acid (HEPES), pH 7.2 with KOH]. Tip resistance ranged from 3 to 4 MΩ. Isolated cardiomyocytes were maintained in an extracellular solution containing: 140 mM NaCl, 5.4 mM KCl, 1 mM MgCl_2_, 1 mM CaCl_2_, 0.33 mM NaH_2_PO_4_, 5 mM HEPES, 10 mM glucose, 0,001 mM, and nifedipine (pH 7.4 with NaOH). Membrane currents were evoked by application of 400 ms voltage steps between −60 and +60 mV in 10 mV increments from a holding potential of −50 mV and measured using the whole cell configuration. TASK-1 current densities were calculated by subtracting background potassium currents before and after administration of 200 nM A293 as described earlier (Limberg et al., 2011; Schmidt et al., 2014, 2019; Wiedmann et al., 2020a). To assess the effects of altered background potassium currents on atrial actions potentials (APs), isolated atrial cardiomyocytes were studied using the patch clamp technique under current clamp conditions. APs were evoked in current clamp mode with holding current of −40 pA by injection of brief current pulse (2 ms, 1 nA) at 0.2 Hz. Patch pipettes for AP recordings were back-filled with 134 mM K gluconate, 6 mM NaCl, 1.2 mM MgCl_2_,1 mM MgATP, and 10 mM HEPES (pH 7.2 with KOH). The extracellular Tyrode’s solution during current clamp measurements consisted of 137 mM NaCl, 5.4 mM KCl, 2 mM CaCl_2_, 1 mM MgSO_4_, 10 mM glucose, and 10 mM HEPES (pH 7.3 with NaOH). Experiments were performed at room temperature (21–25°C), data were not corrected for liquid junction potentials (11.2 mV for voltage clamp measurements and 15.1 mV for AP measurements, calculated with pClamp10) and no leak subtraction was performed.

### Measurement of A293 Plasma Levels

Blood samples were collected in lithium-heparinized tubes (SARSTEDT AG & Co. KG, Nümbrecht, Germany) every 24 h, directly before the daily application of A293 and during baseline as well as final operations. Due to blood clots in the central venous catheters, which still allowed daily application of A293 but made blood sampling impossible, sample collection remained incomplete in several pigs.

After centrifuging at 2,500 × *g* for 10 min at room temperature, the plasma was stored at −20°C until analysis. Plasma concentrations of A293 were measured by an ultra-performance liquid chromatography coupled to tandem mass spectrometry (UPLC-MS/MS) assay. Prior to the analysis, each plasma sample was spiked with the internal standard A293-d8 (kindly provided by Sanofi-Aventis Deutschland GmbH, Frankfurt, Germany) to compensate for potential sample loses during the analysis. A293 was extracted from plasma via liquid-liquid extraction at pH 9.0 using tert-butyl methyl ether (Merck KGaA, Darmstadt, Germany). Chromatographic separation was achieved with an Acquity UPLC^®^ system (Waters Corporation, Milford, MA, United States) equipped with an Acquity UPLC^®^ BEH C18 column (Waters Corporation) in gradient mode (4.5 min). As eluents a mixture of water (Arium^®^ mini Ultrapure Water Systems, Sartorius AG, Göttingen, Germany), 5% acetonitrile (Biosolve BV, Valkenswaard, Netherlands) and 0.01% formic acid (Biosolve BV) (A) and of acetonitrile with 0.01% formic acid (B) was used. After positive electrospray ionization mass-to-charge transitions of m/z 406.3 > 122.1 (A293) and m/z 414.3 > 122.1 (A293-d8) were monitored by a Xevo TQ-S tandem mass spectrometer (Waters Corporation) for the quantification of A293 concentrations in plasma. The assay was linear over a calibration range of 5 to 2,500 ng/ml. The lower limit of quantification was 5 ng/ml. If plasma concentrations exceeded the calibration range, plasma was diluted 10-fold or 100-fold as necessary using blank plasma and reanalyzed. Plasma concentrations of A293 were determined with an accuracy of 90 and 103% and a maximum precision deviation of 12%.

### Statistical Analysis

Prism 8.0 (GraphPad, La Jolla, CA, United States) and Excel (Microsoft, Redmond, WA, United States) software was used for data acquisition and analysis. Data are expressed as mean ± standard error of the mean (SEM). Student’s *t*-tests (two-tailed tests) were applied to compare statistical significance of the results. In cases of small sample sizes where normality could not be assumed, Wilcoxon matched-pairs tests and Mann–Whitney tests were used. *P* < 0.05 was considered as statistically significant. If the hypothesis of equal means could be rejected at the 0.05-level, pair-wise comparisons of groups were made and the probability values were adjusted for multiple comparisons using the Bonferroni correction. Analysis of variance (ANOVA) was performed before pair-wise comparisons.

## Results

### A293 Protects From AF-Induced Atrial Effective Refractory Period (AERP) Shortening

**FIGURE 2:**
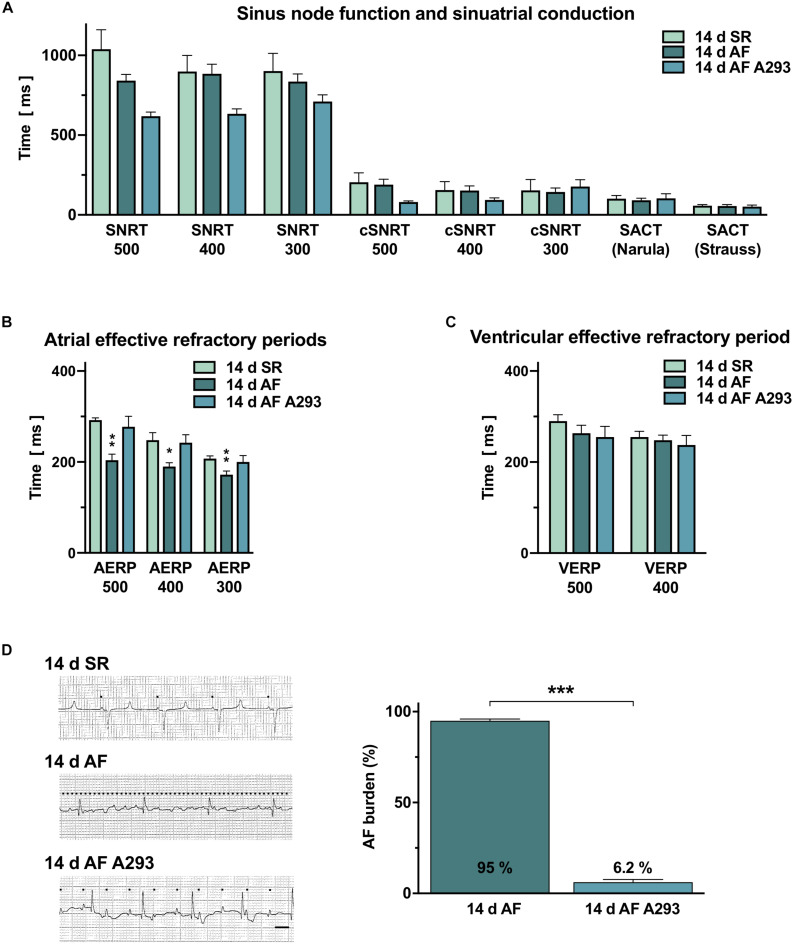
Electrophysiological studies. **(A)** Sinus node recovery times (SNRTs), corrected SNRTs (cSNRTs) and sinoatrial conduction times (SACT), measured according to Strauss et al. (1973) and Narula et al. (1978) compared among the three groups (means of *n* = 4–5 pigs). **(B)** Atrial effective refractory periods (AERPs) were significantly reduced in the atrial fibrillation (AF) induction group and could be partially restored by A293 (*n* = 4–5 pigs). **(C)** No significant differences in ventricular effective refractory periods (VERPs) were observed between the three groups (*n* = 4–5 pigs). **(D)** Left panel: Representative surface ECG recordings from all groups on day 14 (left side). Black dots indicate P waves (scale bar, 200 ms). Right panel: AF burden (i.e., diagnosis of AF in daily surface ECGs in relation to the cumulative number of surface ECGs, documented during the 14 days treatment period) was significantly reduced by A293 treatment (means of *n* = 4–5 animals; error bars, SEM; *P < 0.05 vs. SR; **P < 0.01 vs. SR; ***P < 0.001 vs. 14 days AF from Student’s *t*-tests).

To investigate whether A293 can be used for rhythm control in a porcine model of persistent AF, we performed EP investigations and implanted pacemaker devices in 15 pigs. These were randomized to three groups of 5 animals, two groups with induced AF and one SR control group (Figure 1A). In the two AF groups, AF was induced by repeated episodes of atrial burst-stimulation of the implanted pacemakers and AV node ablations (Supplementary Figure 1) were performed to prevent development of tachycardia-induced heart failure, a common limitation among porcine AF models. One AF group received sham treatment and the other AF group was treated with A293 1 mg/kg bodyweight once per day i.v. as short infusion. In the third group of SR control pigs, burst-pacing was deactivated and pacemakers were set to DDD 50/min mode. Following the treatment period of 14 days, all animals with AF were cardioverted before the second EP investigation. Subsequent to cardioversion, no significant differences were observed in surface ECGs in the three groups (Figure 1B; *n* = 4-5). Notably, PQ intervals could not be recorded after AV node ablations. Due to AV node ablations in animals with induced AF, left ventricular ejection fraction quantified by transthoracic echocardiography did not change over the treatment period (Figure 1C). SNRTs and cSNRTs tended to be reduced by A293 treatment (Figure 2A). Sinuatrial conduction times, measured according to Narula et al. (1978) or Strauss et al. (1973) were not affected (*n* = 4-5 animals each; Figure 2A).

Shortening of atrial refractoriness is a common hallmark of atrial remodeling. As expected, AERP measured at a BCL of 500 ms (AERP_500_) were significantly shortened in the AF group receiving sham treatment relative to the SR control group (AF group with sham treatment: 204 ± 13.3 ms; SR control group: 292 ± 4.9 ms; *P* = 0.0015; Figure 2B). In the AF group receiving A293, the AERP_500_ values were similar to those observed in the SR control group indicating an antiarrhythmic effect (277.5 ± 22.9 ms; *P* = 0.58 vs. SR; Figure 2B). Furthermore, AERP_400_ and AERP_300_ values were comparable between the AF group treated with A293 and the SR control group but reduced in the AF group receiving sham treatment (AERP_400_: SR control group: 248 ± 16.6 ms; AF group receiving sham treatment: 190 ± 8.4 ms [*P* = 0.021 vs. SR]; AF group receiving A293: 242.5 ± 17.5 ms [*P* = 0.83 vs. SR]; AERP_300_: SR control group: 207.4 ± 5.8 ms; AF group receiving sham treatment: 172 ± 8.0 ms [*P* = 0.0082 vs. SR]; AF group receiving A293: 200 ± 14.1 ms [*P* = 0.65 vs. SR]). Of note, no significant changes were observed in ventricular ERP (VERPs) under continuous A293 treatment indicating that no off-target effects were present at the level of ventricular electrophysiology (Figure 2C).

In animals of the AF group receiving sham treatment, AF was present in 95.0 ± 0.9% of the analyzed surface ECG recordings during the treatment period of 14 days (*n* = 56 ECG recordings from 5 animals) (Figure 2D). By contrast, in animals of the AF group receiving A293, AF was present in only 6.2 ± 1.5% of the recorded surface ECGs (*n* = 56 ECG recordings from 5 animals; *P* < 0.0001 vs. AF group receiving sham treatment).

During daily clinical examinations no relevant side effects of A293 were documented. Of note, no proarrhythmogenic side effects of A293 on the ventricular electrophysiology were observed upon pacemaker interrogation at the end of the 14 days treatment period, and it was further not possible to induce arrhythmia during programmed ventricular stimulation. The average plasma trough levels of A293 during the 14 days period, measured by mass spectrometry are visualized in Figure 1D. Please note that blood clots in central vein catheters caused a relevant loss of follow up at some time points. After 4 days of A293 treatment a steady state could be observed.

### Effects of A293 Treatment on the Cellular Electrophysiology of Atrial Cardiomyocytes

**FIGURE 3:**
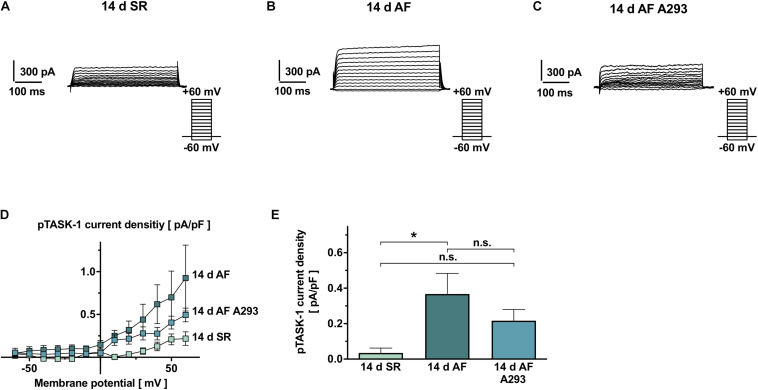
Patch clamp recordings of atrial TASK-1 currents from SR and AF pigs, as well as AF pigs during A293 treatment. **(A–C)** Representative TASK-1 current recordings, performed in atrial cardiomyocytes isolated from atrial fibrillation (AF) or SR pigs at the end of the 14 days treatment period. TASK-1 currents were calculated as differences to background currents after administration of 200 nM A293. **(D)** Current-voltage relationships of isolated TASK-1 current densities for the treatment and control groups (*n* = 10–15 cells, from 4 different animals per group). **(E)** Comparison of average A293-sensitive current densities among the study groups, quantified at the end of a +20 mV pulse (insert: pulse protocols and scale bars; dotted lines, zero current levels; error bars, SEM; *P < 0.05 from Student’s *t*-tests).

To assess the effects of long-term TASK-1 inhibition by A293 at the cellular level, patch clamp measurements were performed in cardiomyocytes isolated from atrial tissue samples. Figures 3A–C shows representative TASK-1 currents recorded from atrial cardiomyocytes of the AF groups receiving sham or A293 treatment and the SR control group. As expected, TASK-1 currents were activated at ≥10 mV and showed open rectification (Goldman-Hodgkin-Katz rectification), a typical feature of K_2__*P*_ currents (Goldstein et al., 2001) (Figure 3D). A293 sensitive current densities, quantified at +20 mV are visualized in Figure 3E. Atrial cardiomyocytes isolated from the AF group receiving sham treatment showed significantly increased TASK-1 currents (0.37 ± 0.12 pA/pF; *n* = 13 cells from 4 individual animals) compared to the SR control group (0.034 ± 0.028 pA/pF; *n* = 15 cells from 4 individual animals; *P* = 0.015). Cardiomyocytes from animals of the AF group treated with A293 showed intermediate TASK-1 currents that were, however, not significantly different in comparison to the SR control group (0.22 ± 0.063 pA/pF; *n* = 10 cells from 4 individual animals; *P* = 0.3 vs. SR control group; Figure 3E).

**FIGURE 4:**
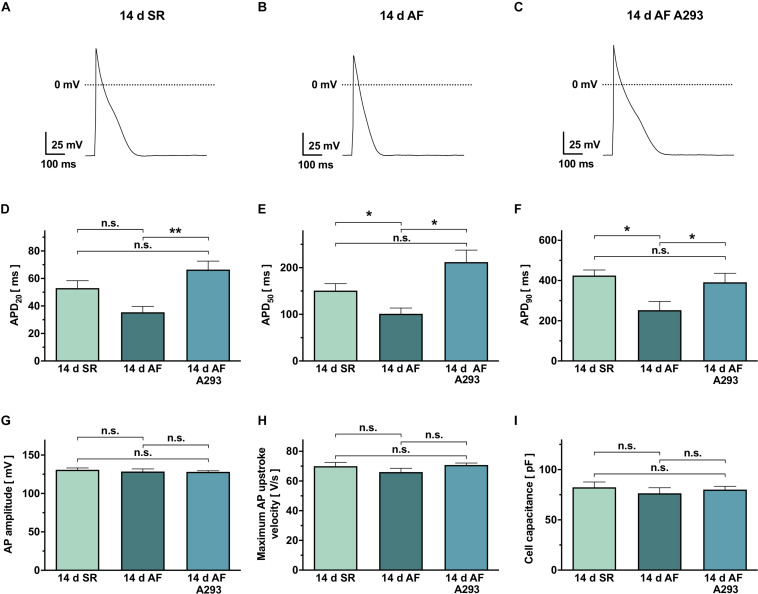
Action potential recordings from isolated atrial cardiomyocytes. **(A–C)** Representative action potential (AP) recordings from atrial cardiomyocytes of the SR control group **(A)**, the atrial fibrillation (AF) group receiving sham treatment **(B)**, and the AF group receiving A293 **(C)**. **(D–F)** Comparison of the corresponding mean AP durations at 20% (APD_20_, **D**), 50% (APD_50_, **E**) or 90% repolarization (APD_90_, **F**). **(G,H)** No statistically significant differences were observed among AP amplitude **(G)** or maximum AP upstroke velocity **(H)**. **(I)** Similar cell capacities were recorded in all 3 study groups. Means of *n* = 4–13 cells isolated from 4 animals per group; error bars, SEM; dashed lines, zero potential levels; *P < 0.05 **P < 0.01 from Student’s *t*-tests.

Figures 4A–C shows representative recordings of atrial APs, elicited by brief current pulse injections (1 nA, 2 ms, 0.2 Hz stimulation rate, holding current of −40 pA). In cardiomyocytes of the AF group receiving sham treatment, APDs at 20% repolarization (APD_20_) showed a numerical tendency toward smaller values (35.4 ± 4.3 ms; *n* = 4 cells from 4 individual animals) when compared to the SR control group (53.0 ± 5.4 ms; *n* = 5 cells from 4 different animals; *P* = 0.036; Figure 4D). In the AF group receiving A293 treatment, APD_20_ values were significantly prolonged relative to the AF group receiving sham treatment (66.6 ± 6.1 ms; *n* = 13 cells from 4 individual animals; *P* = 0.0008; Figure 4D). In the AF group receiving sham treatment, APD_50_ values were significantly smaller (92.20 ± 10.7 ms; *n* = 4 cells from 4 individual animals) compared to the SR control group (150.8 ± 14.9 ms; *n* = 5 cells from 4 different animals; *P* = 0.016; Figure 4E). Among cardiomyocytes of the AF group receiving A293 treatment, APD_50_ values were significantly prolonged relative to the AF group receiving sham treatment (212.0 ± 25.7 ms; *n* = 13 cells from 4 individual animals; *P* = 0.024; Figure 4E). Similarly, APD_90_ values were reduced in the AF group receiving sham treatment (252.6 ± 43.6 ms; *n* = 5 cells from 4 individual animals) relative to the SR control group (425.2 ± 27.2 ms; *n* = 5 cells from 4 animals; *P* = 0.013) and intermediate in the AF group treated with A293 (391.5 ± 44.8 ms; *n* = 13 cells from 4 individual animals; *P* = 0.046 vs. AF group receiving sham treatment; Figure 4F). No significant changes were observed among AP amplitude (Figure 4G) or maximum AP upstroke velocity (Figure 4H). Please note that all cardiomyocytes from the three study groups showed comparable cell capacities (Figure 4I).

**FIGURE 5:**
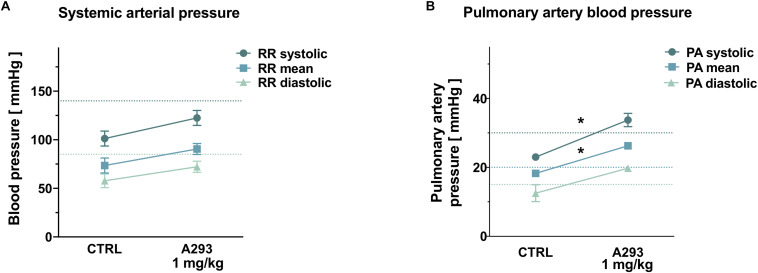
Effects of TASK-1 inhibition on pulmonary artery pressure. **(A,B)** Systemic arterial **(A)** and pulmonary artery **(B)** blood pressure levels of *n* = 4 anesthetized pigs under control conditions (CTRL) and after intravenous application of A293 (1 mg/kg body weight). Means of *n* = 4 animals; error bars, SEM; *P < 0.05 from Student’s *t*-tests; dotted lines mark the normal range in humans.

Taken together, the patch clamp investigations in atrial cardiomyocytes indicated that the long-term A293 treatment reduced the increase of TASK-1 currents and the APD shortening caused by AF.

### Implications of TASK-1 Channel Inhibition on PA Pressure

As TASK-1 channel expression was described in PA smooth muscle cells among different species and TASK-1 currents were discussed to regulate the PA vascular tone, TASK-1 current inhibition might induce an increase in PA pressure. Therefore, to study acute hemodynamic effects of TASK-1 inhibition, 4 anesthetized and mechanically ventilated pigs were treated with intravenous infusion of 1 mg/kg bodyweight A293 and systemic as well as PA pressure values were invasively measured. Mean systemic arterial pressure values showed a numerical, albeit not statistically significant increase from 101.3 ± 7.7/57.8 ± 7.1/73.5 ± 7.7 mmHg (systolic/diastolic/mean blood pressure) to 122.5 ± 7.7/72.3 ± 5.8/90.5 ± 5.7 mmHg (Figure 5A). Administration of A293 increased PA pressure levels from 23.0 ± 0.7/12.5 ± 2.5/18.25 ± 0.9 mmHg (systolic/diastolic/mean blood pressure) to 33.75 ± 1.9/19.8 ± 0.3/26.25 ± 0.3 mmHg (*P*_*sys*_ = 0.0061; *P*_*diast*_ = 0.049; *P*_*mean*_ = 0.0041; Figure 5B).

## Discussion

Using a translationally relevant large animal model, we showed that blockade of atrial TASK-1 currents by A293 exerts antiarrhythmic effects *in vivo* that can be employed for rhythm control of AF. Daily treatment with A293 in pigs with induced AF abrogated the ERP shortening and substantially reduced the AF burden. Patch clamp measurement performed on isolated atrial cardiomyocytes again demonstrated TASK-1 involvement at a cellular level. The observations of reduction of AF-related APD shortening and TASK-1 current upregulation under A293 therapy are in line with previous studies in cardiomyocytes isolated from AF patients (Schmidt et al., 2015, 2017). This result suggests that the applied large animal model of persistent AF has important similarities with the pathophysiology of AF in humans.

From *in vitro* experiments, studying the inhibition of TASK-1 channels by A293 in the heterologous expression system of *Xenopus laevis* oocytes a half-time of the dissociation rate of 2.81 min can be derived (see Supplementary Materials and Supplementary Figure 2). Accordingly, it might be expected that in cardiomyocytes from the A293 treatment group, the TASK-1 inhibition by A293 is reversible during cell isolation and the reduction in isolated TASK-1 current observed in the patch clamp measurements was indeed due to prevention of atrial electrical remodeling under chronic A293 therapy. These observations are in line with TASK-1 expression data, obtained from pigs that underwent a comparable burst-pacing protocol for AF induction (Schmidt et al., 2019). In this study AF-induced upregulation of single cardiomyocyte TASK-1 current was accompanied by a robust increase in atrial TASK-1 mRNA and protein levels (Schmidt et al., 2019).

The observation that A293 had no effect at the level of the ventricles is consistent with the previous finding that the TASK-1 channel is specifically expressed in the atria of the human and the porcine heart (Schmidt et al., 2015, 2019; Wiedmann et al., 2019). Serial echocardiograms excluded effects of TASK-1 inhibition by A293 on the left ventricular ejection fraction.

Previous *in vitro* studies performed by our lab and other groups (Putzke et al., 2007b; Schmidt et al., 2015) determined IC_50_ values of A293 on heterologously expressed TASK-1 channels in the range of 250 nM. At 3 μM A293 a ∼90% TASK-1 inhibition could be observed. IC_50_ values for K_*V*_1.5 were reported to be 10- to 43-fold higher (Wirth et al., 2007; Ehrlich et al., 2008; Kiper et al., 2015). At a concentration of 1 μM TASK-3 was in addition to TASK-1 the only channel to display significant inhibition among all functional members of the K_2__*P*_ family and several other cardiac potassium channels (Putzke et al., 2007b; Schmidt et al., 2015). Of note, the TASK-3 channel did not show cardiac expression (Schmidt et al., 2015). In the concentration range of 30–50 μM increasing off-target effects on I_*Kr*_, I_*KACh*_, and I_*L–Ca*_ channels were observed (Wirth et al., 2007). Taking into account these preliminary studies, the A293 plasma levels measured in our work are essentially within the range where TASK-1 specificity can be assumed. The fact that, however, no information about plasma protein binding of A293 is available makes a direct comparison of plasma levels measured in our study pigs and *in vitro* data difficult.

Consistent with our results, two previous studies had shown that A293 prolongs the AERPs in healthy pigs and that it can be used for pharmacological cardioversion of artificially induced acute episodes of AF in a porcine model (Wirth et al., 2007; Wiedmann et al., 2020a). Here, we showed that long-term administration of A293 can further be successfully used for rhythm control of persistent AF.

Among atrial-specific ion channels, the TASK-1 channel represents a promising target for anti-arrhythmic therapy because it is upregulated in AF. The aromatic carbonamide A293 is a TASK-1 channel inhibitor with high affinity which could therefore serve as an anti-arrhythmic drug (Putzke et al., 2007b). A study in rats showed that radioactively labeled A293 (10 mg/kg bodyweight, oral intake) crossed the blood–brain-barrier only to a minor degree (Bittner et al., 2012). This is important because TASK-1 is expressed throughout the central nervous system and inhibition of neuronal TASK-1 currents might cause neurological side effects. Consistently, in this study, no neurological side effects were observed.

When measuring the acute effect of A293 on hemodynamic parameters, however, a significant increase in systolic PA-pressure values from 23.0 to 33.75 mmHg could be observed, while systemic arterial pressure values only showed a mild increase. TASK-1 expression was described in human PA smooth muscle cells (Olschewski et al., 2006). In these cells, TASK-1 currents are crucial for setting the basal membrane potential and consecutively regulating pulmonary vascular tone. Further, TASK-1 loss-of-function mutations were identified in patients suffering from idiopathic PA hypertension (Ma et al., 2013). Experimental data regarding the effects of TASK-1 inhibition on the PA vascular tone remain sparse and partially contradicting. While rats were reported to exhibit elevated PA pressure levels after chronic exposure to a TASK-1 inhibitor (Antigny et al., 2016), TASK-1 knockout mice displayed PA and right ventricular pressure levels that did not differ from their wild-type littermates (Kitagawa et al., 2017; Murtaza et al., 2017). This is to our best knowledge the first report studying hemodynamic effects of TASK-1 inhibition in a clinically relevant large animal model. Even though the clinical significance of this rather mild increase remains uncertain, elevation of PA pressure might constitute a major limitation of systemic TASK-1 channel inhibition. Since A293 does not cross the blood–brain barrier, it is unlikely that the observed effect is due to interaction with central nervous TASK-1 channels, favoring the hypothesis of direct TASK-1 inhibition in PA smooth muscle cells. Further studies will be necessary to clarify whether a range of TASK-1 plasma levels exists where antiarrhythmic effects of TASK-1 inhibition can be utilized without clinically relevant side effects on PA pressure levels.

There are several limitations of this study. Sample sizes were relatively small due to regulations on animal protection. In the present investigation we studied TASK-1 currents or APs not in multicellular preparations but only in isolated cardiomyocytes. Further, the whole cell configuration was used which might disturb the physiologic intracellular milieu. Finally, APs and currents were recorded at room temperature in our study and their regulation may differ at physiological temperature. The porcine model only indirectly resembles AF. The animal model might lack components of atrial cardiomyopathy such as heart failure that is caused by tachymyopathy. In pigs, the development of a tachymyopathy was avoided by AV node ablation. However, in our opinion this reflects the situation in the majority of AF patients, as tachymyopathy is only present in a subset of AF patients. As the investigators performed interventions as well as daily follow-ups they were not blinded.

Our large animal study constitutes the first proof-of-concept that the inhibition of TASK-1 ion channels can be used for rhythm control of persistent AF. Further clinical investigations are, however, mandatory to assess whether TASK-1 inhibitors are applicable for treatment of AF in patients. For acute cardioversion of paroxysmal or persistent AF, the DOCTOS trial (doxapram conversion to SR; EudraCT No: 2018-002979-17) currently evaluates whether TASK-1 inhibition might serve as a strategy for AF treatment. In this trial, the FDA- and EMA-approved respiratory stimulant doxapram that was recently identified as potent TASK-1 blocker is administered to patients with paroxysmal or persistent AF (Cotton, 2013; Chokshi et al., 2015; O’Donohoe et al., 2018; Cunningham et al., 2019; Rödström et al., 2020). In contrast to doxapram, A293 does not cross the blood–brain barrier and might therefore have the advantage of not causing central nervous side effects.

## Conclusion

Taken together, our translational *in vivo* study could demonstrate that long-term A293 treatment prolongs atrial refractoriness without relevant alterations of ventricular repolarization. These results confirm the role of TASK-1 as a promising drug target in AF and therefore hopefully promote the translation of a novel, mechanism-based antiarrhythmic paradigm into clinical practice. As TASK-1 inhibition, however, was shown to induce a significant increase in PA pressure, further studies are warranted to clarify its safety in chronic use.

## Data Availability Statement

The raw data supporting the conclusions of this article will be made available by the authors, without undue reservation.

## Ethics Statement

All animal experiments have been carried out in accordance with the Guide for the Care and Use of Laboratory Animals as adopted and promulgated by the United States National Institutes of Health (NIH publication No. 86-23, revised 1985), with the EU Directive 2010/63/EU, and with the German Law on the Protection of Animals. Approval for experiments involving pigs was granted by the local Animal Welfare Committee (Regierungspräsidium Karlsruhe, Germany, reference numbers G-296/14 and G-217/18). Approval for experiments involving *Xenopus laevis* was granted by the local Animal Welfare Committee (Regierungspraesidium Karlsruhe, Germany, reference numbers G-221/12 and G-165/19).

## Author Contributions

FW and CS designed the study and co-wrote the manuscript. MB, WH, and NF provided valuable support on experimental design, data analysis and interpretation. FW, CB, and CS performed the large animal experiments. X-BZ, IE-B, and SL performed and analyzed the patch clamp experiments in porcine atrial cardiomyocytes. WH, KF, and MK performed mass spectrometry for assessment of A293 plasma levels. MB, WH, and NF supported manuscript and figure preparation and supervised the project. All authors have approved the submitted version of the manuscript and have agreed to be personally accountable for their contributions.

## Conflict of Interest

FW and CS have filed a patent application for KCNK3-based gene therapy for cardiac arrhythmia. FW, WH, and CS have filed a patent application for pharmacological TASK-1 inhibition in treatment of atrial arrhythmia. A293-d8 was kindly gifted by Sanofi-Aventis Deutschland GmbH (Frankfurt, Germany). The remaining authors declare that the research was conducted in the absence of any commercial or financial relationships that could be construed as a potential conflict of interest.

## Abbreviations

AERP: atrial effective refractory period
AF: atrial fibrillation
AP: action potential
APD: action potential duration
AV node: atrioventricular node
BCL: basic cycle length
cAF: chronic atrial fibrillation
cSNRT: corrected sinus node recovery time
DMSO: dimethyl sulfoxide
ECG: electrocardiogram
EP: electrophysiology
ERP: effective refractory period
i.m.: intramuscular
i.v.: intravenous
K_2__*P*_: two-pore-domain potassium
PA: pulmonary artery
RF: radiofrequency
SACT: sinoatrial conduction time
SNRT: sinus node recovery time
SR: sinus rhythm
TASK: TWIK-related acid-sensitive K^+^ channel
TWIK: tandem of P domains in a weak inward rectifying K^+^ channel
UPLC-MS/MS: ultra-performance liquid chromatography coupled to tandem mass spectrometry
VERP: ventricular effective refractory period.
