# Supplementary material for: The Experimental TASK-1 Potassium Channel Inhibitor A293 Can Be Employed for Rhythm Control of Persistent Atrial Fibrillation in a Translational Large Animal Model
Source: Front Physiol. 2021 Jan 21;11:629421. doi: 10.3389/fphys.2020.629421 (PMC7858671; doi:10.3389/fphys.2020.629421)
Supplement: Supplementary file 1 [file Table_1.DOCX]

Supplementary Material

## Supplementary Methods

### Animal ethics

Animal experiments were carried out in accordance with the Guide for the Care and Use of Laboratory Animals as adopted and promulgated by the U.S. National Institutes of Health (NIH publication No. 86-23, revised 1985), with EU Directive 2010/63/EU, and with the current version of the German Law on the Protection of Animals. Approval for experiments involving *Xenopus laevis* was granted by the local Animal Welfare Committee (Regierungspraesidium Karlsruhe, Germany, reference numbers G-221/12 and G-165/19).

### Molecular biology

Plasmid constructs containing cDNA encoding human TASK-1 (GenBank accession no. NM_002246) cloned in the expression vector pRAT were kindly provided by Steve Goldstein (Brandeis University, Waltham, MA, USA). The pMAX–pTASK-1 construct, encoding for porcine TASK-1 (KF182337.1), was cloned from porcine cardiac cDNA as described earlier (Schmidt et al., 2014). *In vitro* synthesis of copy RNA (cRNA) was performed, using the mMESSAGE mMACHINE T7 Transcription Kit (Thermo Fisher Scientific Waltham, MA, USA) according to the manufacturer’s instructions. Integrity of transcripts was assessed via agarose gel electrophoresis and concentration was determined by spectrophotometry (ND-2000, Thermo Fisher Scientific).

### *Xenopus laevis* handling

For oocyte preparation, ovarian lobes were surgically removed in aseptic techniques from female *Xenopus* *laevis* frogs (Xenopus Express, Le Bourg, France), anesthetized with tricaine solution (1 g/l, pH 7.5, 15 °C). Frogs were kept in specific pathogen-free aquarium facilities with continuous water flow (Aqua Schwarz GmbH, Göttingen, Germany) at a housing density according to directive 2010/63/EU. Water temperature was kept at 18 °C and room lighting had a light/dark cycle of 12/12 h. Frogs were fed daily and environmental enrichment was provided with huts and PVC pipes. No more than four operations were performed on one individual frog. After collagenase treatment, stages V and VI defolliculated oocytes were manually selected under a stereo microscope and injected with 46 nl nuclease free H_2_O, containing 1.5 ng (hTASK-1) or 25 ng (pTASK-1) cRNA. Measurements were performed 48 h to 72 h after injection of cRNA.

### Two-electrode voltage clamp electrophysiology

Two-electrode voltage clamp recordings of macroscopic potassium currents were performed 1 to 4 d after cRNA injection using a Warner OC-725C amplifier (Warner Instruments, Hamden, CT, USA) and pCLAMP10 (Axon Instruments, Foster City, CA, USA) software for data acquisition. Electrodes were pulled from borosilicate glass (GB 100F-10, Science Products, Hofheim, Germany) using a Flaming/Brown P-87 micropipette puller (Sutter Instruments, Novato, CA, USA). After back-filling with 3 M KCl, tip resistances yielded 0.8 to 2.0 MΩ. All recordings were performed at room temperature (21 to 25 °C) in a standard physiological extracellular solution, consisting of: 101 mM NaCl, 4 mM KCl, 1.5 mM CaCl_2_, 2 mM MgCl_2_, and 10 mM 4-(2-hydroxyethyl)piperazine-1-ethanesulfonic acid ( HEPES; pH 7.4). TASK-1 currents were evoked by application of 500 ms test pulses from ‑ 140 to + 60 mV in 20 mV increments from a holding potential of ‑ 80 mV and quantified at the end of the + 20 mV pulse. Leak currents were not subtracted.

### Statistical *analysis*

PCLAMP 10 (Axon Instruments, Foster City, USA), Origin 8 (OriginLab, Northampton, MA, USA) and Prism 8.0 (GraphPad, La Jolla, CA, USA) software was used for data acquisition and analysis. Data are expressed as mean ± standard error of the mean (SEM). Half-time of the dissociation rate was calculated according to the equation: $Y(x)=Y_{0}+(Plateau- Y_{0})(1^{-\left( -K*x \right)})$, where Plateau is the *Y* value at infinitive times, *K* is the rate constant. The time constant τ was calculated as the reciprocal of *K* and the half-time as ln(2)/*K*.

## Supplementary Results

### Assessing the half-time of the dissociation rate of A293 inhibition in heterologously expressed TASK-1 channels

Following heterologous expression in *Xenopus laevis* oocytes, human and porcine TASK-1 currents were studied using the two-electrode voltage clamp technique. Currents were elicited by application of depolarizing test pulses (500 ms), applied in 20 mV increments from a holding potential of - 140 mV to + 60 mV (0.2 Hz) and current amplitudes were measured at the end of the + 20 mV pulse. After a control period with no significant amplitude changes (12 min), administration of A293 (0.5 µM or 0.05 µM, 20 min) resulted in a fast and time-dependent reduction of outward potassium currents (Supplementary Figure 2A-C). Inhibitory effects of A293 were partially reversible (Supplementary Figure 2A-B and D). Current levels of hTASK-1 reached 91.6 ± 50.4 % (0.05 µM; n = 4) and 90.4 ± 21.9 % (0.5 µM; n = 5) of respective controls 20 min after removal of the drug. Currents of pTASK-1 displayed a reversibility of TASK-1 current inhibition upon washout to 94.4 ± 16.1 % (0.05 µM; n = 3) and 78.2 ± 5.1 % (0.5 µM; n = 3) of respective control measurements. After fitting the washout curve of pTASK-1 and 0.5 µM A293 (the condition that most closely reflects the conditions of our study) with a single exponential equation, a half-time of the dissociation rate of 2.81 min (95 % CI 1.3 - 8.2 min) with τ = 4.1 min (95 % CI: 1.87 – 11.8 min) was calculated. The observation that reversibility of the A293 blockade remains incomplete might be related to intracellular accumulation of the drug to due interactions with the lipophilic yolk of *Xenopus laevis* oocytes. This would however at best lead to an underestimation of reversibility.


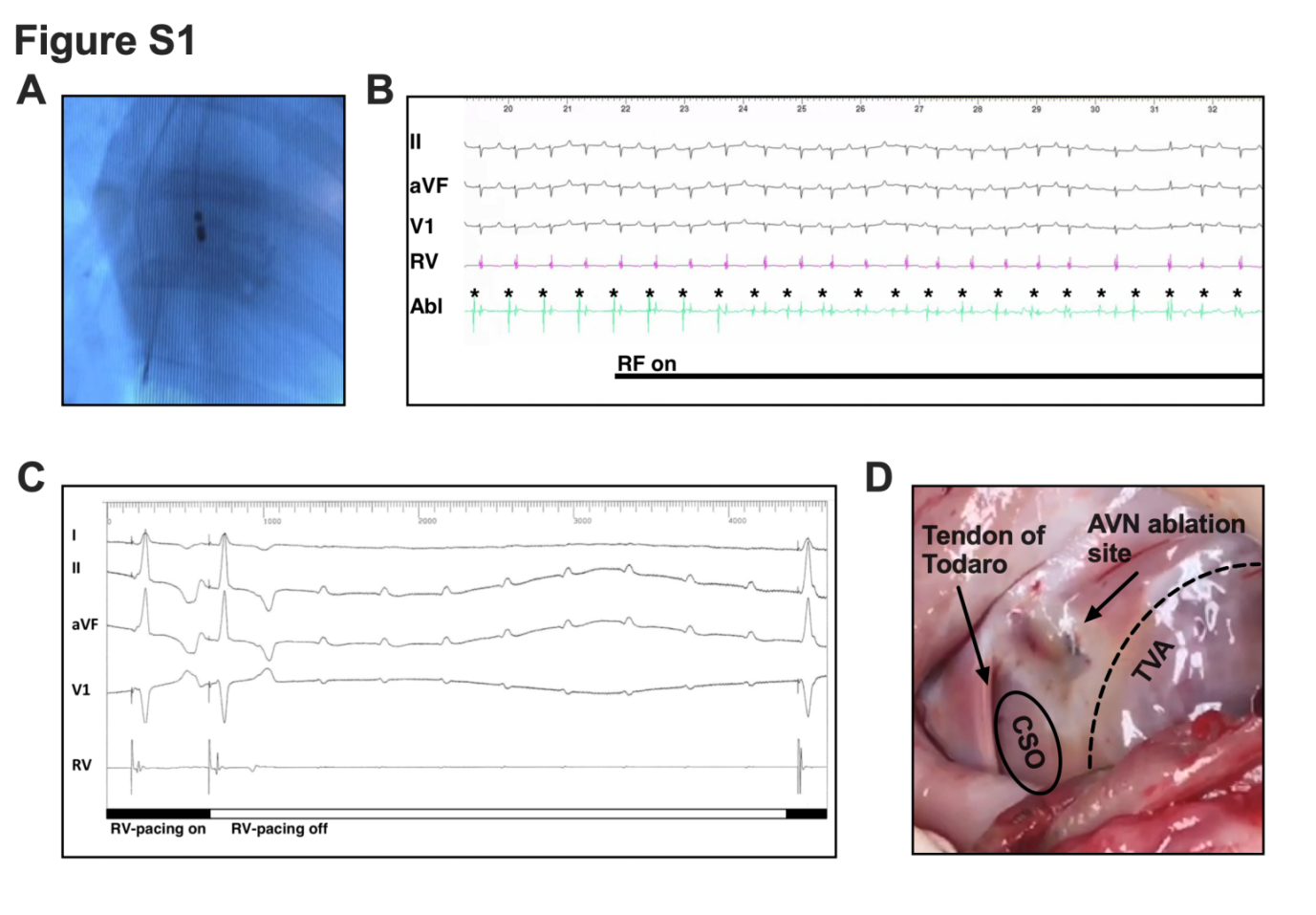


**Supplementary Figure 1. Radiofrequency ablation of the AV node**

***(A)*** Periprocedural fluoroscopy during AV node ablation performed in anteroposterior projection (because of a different rotation of the heart axis in pigs, this corresponds approximately to a left anterior oblique 60° projection in humans). A quadripolar stimulation catheter is located in the right ventricular outflow tract and the ablation catheter is positioned at the AV node. ***(B)*** Visualization of surface ECG leads II, aVF and V1 as well as intracardiac signals (RV, right ventricle; Abl, ablation catheter) during AVN ablation. At the beginning of the trace every ventricular beat is preceded by an atrial beat (marked with an asterisk). Approximately 2 seconds after starting the radiofrequency ablation (RF on, black bar) a dissociation of atrial activity and the junctional escape rhythm can be observed. ***(C)*** Visualization of surface ECG leads I, II, aVF and V1 as well as a signal derived from the ventricular stimulation catheter. Under deactivation of RV pacing asystole for 3.5 s due to complete AV block III° can be observed. ***(D)*** Necropsy 14 days after AVN ablation. The right atrium was opened from the lateral site. Anatomic structures defining the triangle of Koch are visualized (CSO, coronary sinus ostium; TVA, tricuspid valve annulus; AVN, AV node). A distinct post-ablation scar in the region of the compact AV node can be noted.


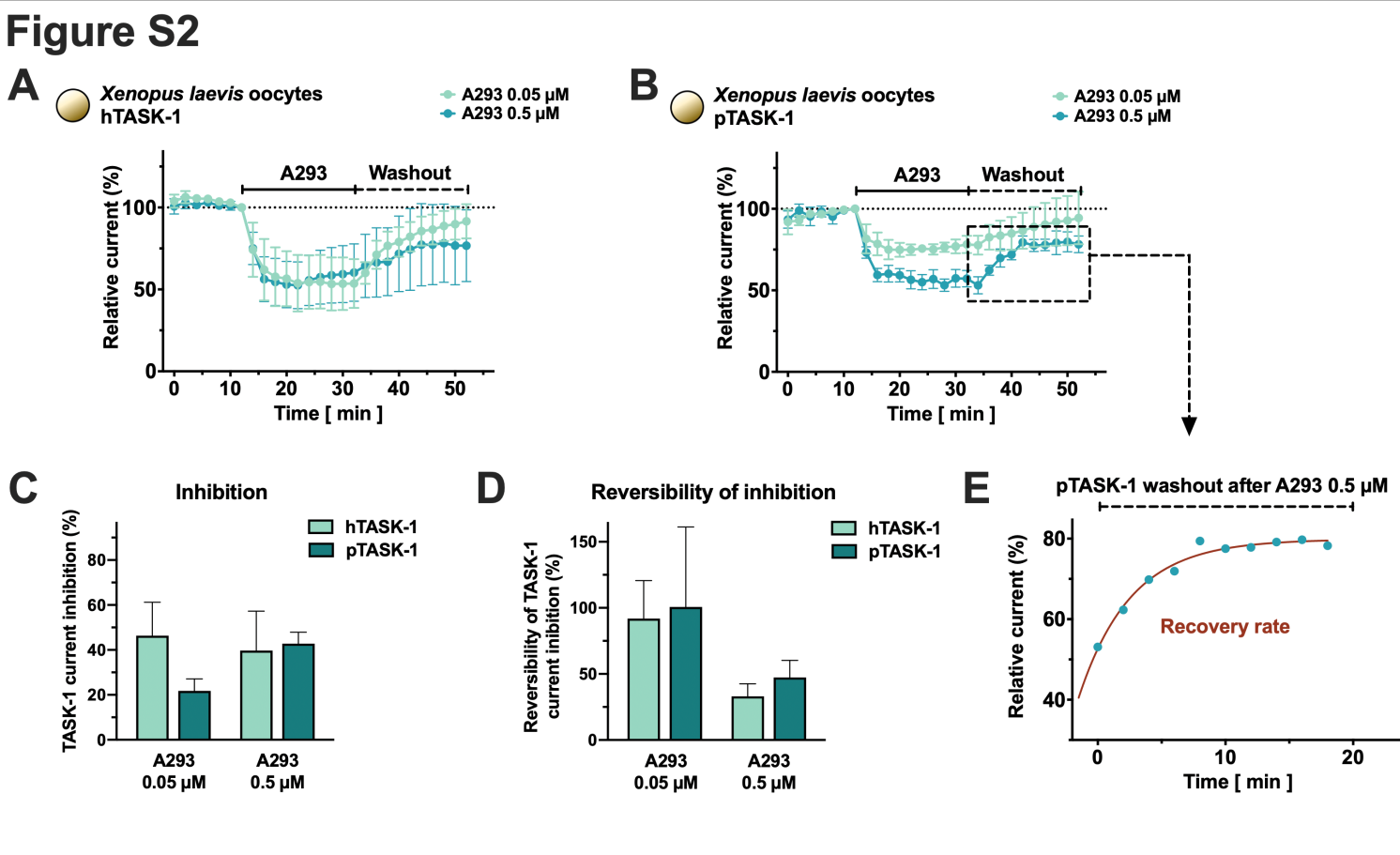


**Supplementary Figure 2. Reversibility of TASK-1 current inhibition by A293**

Two-electrode voltage clamp measurements were performed on human (hTASK-1, ***A***) and porcine (pTASK-1, ***B***) orthologs of the TASK-1 channel, heterologously expressed in *Xenopus* *laevis* oocytes to assess inhibition upon A293 administration and its reversibility. ***(A-B)*** Time-dependent blockade of hTASK-1 ***(A)*** and pTASK-1 ***(B)*** upon application of A293 (0,05 µM or 0,5 µM as indicated; 20 min) and its reversibility upon washout. Data are given as means of n = 3 - 5 cells each ± SEM. ***(C)*** Quantification of TASK-1 current inhibition at the end of the 20 min incubation time. ***(D)*** Reversibility of TASK-1 current inhibition after a 20 min washout period. ***(E)*** By fitting the washout curve of pTASK-1 /0.5 µM A293 with a single exponential, a half-time of the dissociation rate of 2.81 min (95 % CI 1.3 - 8.2 min) was calculated.
